# The Beneficial Effects of Berberine on Brain Functions in Age‐Related Neurological Disorders: From Molecular Signaling to Treatment

**DOI:** 10.1002/fsn3.70563

**Published:** 2025-08-01

**Authors:** Xiaolan Wang, Ruiliang Hou, Zhihao Chen, Xiaoyang Wang, Melika Malek, Haoyu Wang

**Affiliations:** ^1^ Medical College Xijing University Xi'an Shaanxi China; ^2^ Department of Orthopedics, the Second Affiliated Hospital Xi'an Jiaotong University Xi'an Shaanxi China; ^3^ Shahid Beheshti University of Medical Sciences Tehran Iran

**Keywords:** age‐related neurological disorders, berberine, therapy

## Abstract

Berberine (BBR), a naturally occurring compound with diverse medicinal properties, is emerging as a compelling candidate for managing age‐related central nervous system conditions. Preclinical studies have extensively explored its impact on neurodegenerative diseases such as Parkinson's disease, Alzheimer's disease, and Huntington's disease, revealing its capacity to modulate key pathological processes. Mechanistically, BBR is shown to mitigate neuroinflammation, oxidative stress, and endoplasmic reticulum stress, thereby reducing neuronal damage and apoptosis. While these mechanistic insights and preclinical data are robust, clinical evidence remains nascent, necessitating further rigorous investigation to fully understand BBR's therapeutic efficacy and translate its promise into clinical practice. This narrative review provides a comprehensive examination of BBR's application in treating neurological conditions, emphasizing its molecular signaling pathways and critically evaluating the current translational landscape. It also identifies promising directions for future research into BBR's neuroprotective roles.

## Graphical abstract

The positive influence of berberine on the nervous system can be attributed to its diverse effects on multiple targets. The effects of berberine include its antioxidant, anti‐apoptotic, anti‐necroptotic, and anti‐inflammatory properties, as well as its ability to induce autophagy. It was proved that the effectiveness of berberine in treating neurological disorders is through enhancing its brain targeting and bioavailability.

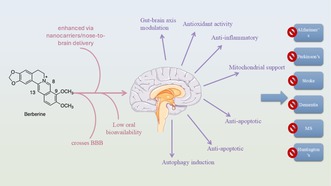

**Abbreviations**
6‐OHDA: 6‐hydroxydopamine
AD: Alzheimer's disease
AIS: acute cerebral ischemic stroke
Akt: protein kinase B
AMPK: AMP‐activated protein kinase
APP: amyloid precursor protein
Aβ: amyloid β
BBB: blood–brain barrier
BBR: berberine
BDNF: brain‐derived neurotrophic factor
BH4: tetrahydrobiopterin
CAT: catalase
ChE: cholinesterase
COMT: catechol‐O‐methyltransferase
COX‐2: cyclo‐oxygenase‐2
CREB: cyclic AMP response element‐binding protein
CTS‐NLCs: Chitosan‐coated nanostructured lipid carriers
CVD: cardiovascular diseases
CYP: cytochrome P450
DA: dopamine
DCs: dendritic cells
dhBBR: dihydroberberine
DOPAC: dihydroxyphenylacetic acid
DOX: doxorubicin
EAE: experimental autoimmune encephalomyelitis
EAM: experimental autoimmune myocarditis
eEF2: eukaryotic elongation factor 2
ELISA: enzyme‐linked immunosorbent assay
ERK: extracellular signal‐regulated kinase
Exos: exosomes
GPx: glutathione peroxidase
GSH: glutathione
GSK3 beta: glycogen synthase kinase‐3 beta
HD: Huntington's disease
HGHI: high glucose and insulin
HIF‐1: hypoxia‐inducible factor‐1
HMGB1: high mobility group box 1
HVA: homovanillic acid
IL: interleukin
IMT: intima‐media thickness
iNOS: inducible nitric oxide synthase
IR: insulin resistance
IUPAC: International Union of Pure and Applied Chemistry
JAK: janus kinase
LINC00943: long intergenic non‐coding RNA 00943
LPS: lipopolysaccharide
LRRK2: leucine‐rich repeat kinase 2
MAO‐A: monoamine oxidase A
MAO‐B: monoamine oxidase B
MAPKs: mitogen‐activated protein kinases
MCAO: middle cerebral artery occlusion
MDA: malondialdehyde
MMP‐9: matrix metalloproteinase‐9
MPTP: 1‐methyl‐4‐phenyl‐1,2,3,6‐tetrahydropyridine
MS: multiple sclerosis
NF‐kappaB: nuclear factor‐kappa B
NFTs: neurofibrillary tangles
NMDA: N‐methyl‐D‐aspartate
NQO1: NAD(P)H quinone oxidoreductase 1
NRF1/2: nuclear factor (erythroid‐derived 2)‐like 1/2
PD: Parkinson's disease
PET: positron emission tomography
PGC‐1alpha: peroxisome proliferator‐activated receptor gamma coactivator 1‐alpha
PI3K: phosphoinositide 3‐kinase
PKC epsilon: protein kinase C epsilon
PPARs: peroxisome proliferator‐activated receptors
ROS: reactive oxygen species
SCFAs: short‐chain fatty acids
SCI: spinal cord injury
SOD: superoxide dismutase
STAT: signal transducer and activator of transcription
SULTs: sulfotransferases
TBI: traumatic brain injury
TH: tyrosine hydroxylase
TLR4: toll‐like receptor 4
TNF‐α: tumor necrosis factor‐alpha
UGTs: UDP‐glucuronosyltransferases
WHO: World Health Organization

## 1 Introduction

Berberine (BBR) and with the IUPAC name of 16,17‐dimethoxy‐5,7‐dioxa‐13‐azoniapentacyclo [11.8.0.02,10.04,8.015,20] henicosa‐1(13),2,4(8),9,14,16,18,20‐octaene and PubChem CID: 2353, is an isoquinoline quaternary alkaloid. It has a molar weight of 336.36 g/mol and can be found in different medicinal herbs such as *Xanthorhiza simplicissima* , *Hydrastis canadensis, Coptis japonica, Coptis chinensis*, and *Berberis aristata* (Ortiz et al. 2014; Cicero and Baggioni 2016; Feng et al. 2019). Yellow powder BBR is not very soluble in ethanol or methanol (Kong et al. 2004; Wang, Feng, et al. 2017). According to sources, BBR is extensively utilized in numerous ancient healing systems, such as Chinese medicine, Ayurveda, and Iranian traditional medicine (Cicero and Baggioni 2016; Kunwar et al. 2006) and it has been used in some cases like diabetes, cancer, hypertension, cardiovascular diseases, Alzheimer's disease (AD), etc. (Wang, Feng, et al. 2017; Imenshahidi and Hosseinzadeh 2016; de Oliveira et al. 2019). Extensive studies have provided evidence that BBR can effectively treat a range of disorders affecting the central nervous system, such as AD, depression, epilepsy, cerebral ischemia, anxiety, and schizophrenia. However, it is important to address these results that have only been observed in animal models in preclinical studies (Fan et al. 2017; Liu et al. 2017; Sedaghat et al. 2017; Fan, Zhang, et al. 2019; Yuan et al. 2019; Rezaeian et al. 2020; Qiu et al. 2022; Zhao, Li, et al. 2021; Kulkarni and Dhir 2010).

The investigation revealed that BBR possesses the ability to diminish the cognitive decline induced by doxorubicin (DOX). Further investigations uncovered that BBR exhibited antioxidant characteristics through the reduction of pro‐inflammatory and cell death‐inducing components, along with NF‐κb, while simultaneously boosting the levels of PGC‐1α and manganese superoxide dismutase. Moreover, BBR can regulate the CREB and BDNF to better regulate the alterations in neuronal connections that arise (Shaker et al. 2021). Furthermore, current studies suggest that BBR has the ability to impede the function of four significant enzymes involved in the progression of AD: acetylcholinesterase, butyrylcholinesterase, and both forms of monoamine oxidase (MAO‐B and MAO‐A) (Yuan et al. 2019). At present, there is a lack of research examining the impact of BBR on diseases affecting the nervous system, and additional studies are necessary to fully comprehend its potential advantages. As the elderly population has grown in recent times, diseases associated with aging, namely neurodegenerative conditions, have also become more prevalent and present a major risk to human well‐being (Heemels 2016; Singh et al. 2024; Checkoway et al. 2011; Prajapati et al. 2025). As stated by the WHO, AD is ranked in the top ten leading causes of death worldwide. In 2019, it ranked third in term1‐s of mortality rates in both the Americas and Europe (Organization WH 2020). Furthermore, conditions that lead to the decline of brain function, such as PD and AD, not only have a considerable impact on those affected and their loved ones, but also pose a pressing public health issue that must be addressed. Degenerative disorders of the nervous system are marked by a gradual breakdown of synapses, glial cells, and neuron functioning (Marchesi et al. 2021; Pathak et al. 2022). The study of autophagy processes, genetic instability, inflammation, and protein clumping is currently a prominent area of research in understanding the causes of neurodegenerative disorders (Hou et al. 2019). Despite considerable advances in studying the development of neurodegenerative disorders like Huntington's, Alzheimer's, Parkinson's, and ALS, as well as the discovery of numerous drugs with neuroprotective properties in cells and animal tests, there has yet to be a medication that can create significant clinical improvements in the underlying progression of these diseases. A medication must be promptly created to address neurodegenerative illnesses while causing minimal adverse reactions. While several reviews have explored BBR's general pharmacological properties or its effects in specific neurological conditions, this narrative review uniquely offers a comprehensive and integrated perspective on BBR's multifaceted beneficial effects across a broad spectrum of age‐related neurological disorders. Unlike reviews focusing solely on preclinical data or a single disease, this work systematically consolidates the most recent findings from both molecular signaling and clinical trial perspectives, emphasizing the translational challenges and opportunities (Ahmed et al. 2015; Imenshahidi and Hosseinzadeh 2016; Kumar et al. 2015). Specifically, we provide an in‐depth analysis of BBR's mechanisms of action—including its impact on neuroinflammation, oxidative stress, mitochondrial dysfunction, and specific molecular pathways (e.g., PI3K/Akt, AMPK, NF‐κB, lncRNAs)—as they relate to various neurodegenerative conditions such as Alzheimer's, Parkinson's, multiple sclerosis (MS), stroke, spinal cord injury (SCI), Huntington's, and other forms of dementia. Furthermore, this review distills the current evidence from emerging clinical investigations, critically discussing the progress and the gaps in translating preclinical success to human therapeutic applications. By highlighting these specific advancements and ongoing challenges, this review serves as a timely resource to guide future research directions and accelerate the development of BBR‐based strategies for age‐related neurological disorders.

## 2 BBR Structure and Biological Function

**FIGURE 1:**
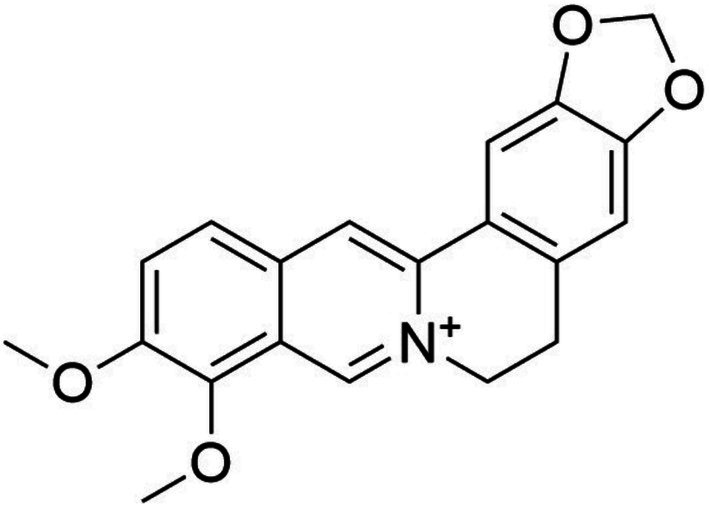
Chemical structures of berberine.

BBR is a type of alkaloid known as isoquinoline, commonly found in numerous medicinal plants, primarily in those of the Berberis species (such as *Berberis vulgaris* L. from the Berberidaceae family). Another instance of this phenomenon can be observed in *Coptis chinensis* Franch., a species from the Ranunculaceae family. In traditional Chinese medicine, this plant is utilized for its anti‐bacterial, anti‐diarrheal, anti‐protozoal, and anti‐fungal properties, often in conjunction with other medicinal herbs (Mirska et al. 1972; Yamamoto et al. 1993). Figure 1 depicts the complete chemical composition of BBR. Over time, increasing proof has unveiled a diverse range of physiological effects of BBR, including but not limited to antiviral, antibacterial, and anti‐inflammatory properties (Imanshahidi and Hosseinzadeh 2008; Kuo et al. 2004).

**FIGURE 2:**
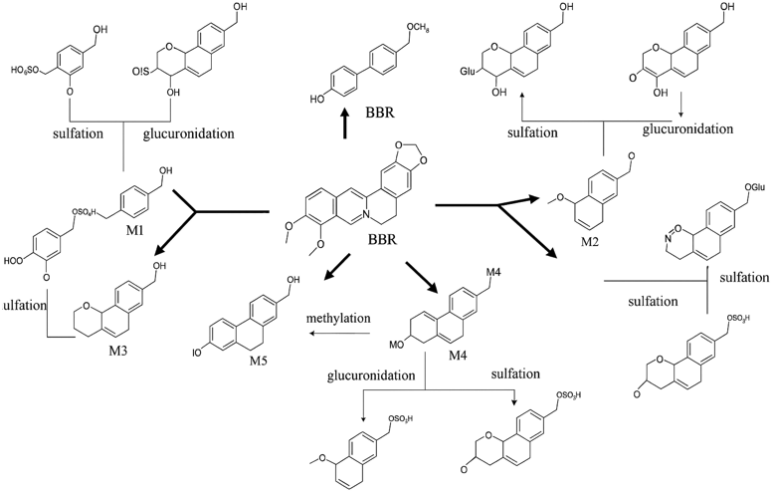
Metabolic pathway of BBR.

Following the intake of BBR, the gastrointestinal systems of rats, mice, beagle dogs, hamsters, and rabbits efficiently assimilated this compound through oral administration (Yamamoto et al. 1993; Imanshahidi and Hosseinzadeh 2008; Kuo et al. 2004; Shanbhag et al. 1970); however, the bioavailability was quite low (below 1%) (Kuo et al. 2004; Yu et al. 2011). Next, the primary distribution of BBR was to the liver, with secondary distribution to various other organs like the intestines, muscles, lungs, pancreas, kidneys, brain, and heart (Imanshahidi and Hosseinzadeh 2008; Durairajan et al. 2012). Notably, BBR can cross the BBB (Imanshahidi and Hosseinzadeh 2008). Despite being orally administered, the level of BBR found in the brain was considerably low, at approximately 1 nanogram per gram of brain tissue (Imanshahidi and Hosseinzadeh 2008). After that, the BBR that was taken in underwent a series of reactions in phase I metabolism, which involved demethylenation, demethylation, and reduction, ultimately resulting in the creation of metabolites M1‐6 (Imanshahidi and Hosseinzadeh 2008; Feng et al. 2015). The first stage of metabolites was typically regulated by specific P450 enzymes, specifically CYP2D6, CYP3A4, and CYP1A2 (He et al. 2017). Moreover, according to documented evidence, the nitroreductase enzyme present in the gut microbiota has the ability to transform BBR into a readily absorbable form called dihydroberberine (dhBBR) (Feng et al. 2015). Metabolites in phase II were produced through the processes of sulfation, methylation, and glucuronidation of metabolites from phase I, using enzymes such as sulfotransferases (SULTs), catechol‐O‐methyltransferase (COMT), and UDP‐glucuronosyltransferases (UGTs) (Chen et al. 2017; Huang et al. 2017). The majority of BBR and its by‐products were expelled through the digestive system, with a smaller portion being eliminated through bile and urine (Chen et al. 2017). An overview of BBR's metabolic attributes is depicted in Figure 2 (Wang, Feng, et al. 2017).

## 3 BBR and Neurological Disorders

One of BBR's most notable strengths is its ability to bypass the blood–brain barrier (BBB) and provide strong protection against various neurodegenerative disorders such as cerebral ischemia, Parkinson's disease (PD), depression, anxiety, AD, and schizophrenia (Shayganfard 2023; Song et al. 2020; Wang, Sheng, et al. 2021; Wang et al. 2023; Wu et al. 2022; Zhang, Li, et al. 2017). New research reveals that BBR consumption can effectively address harmful effects on the nervous system caused by a range of factors. These include drugs like DOX and 6‐OHDA, environmental toxins such as chlorpyrifos, mercury, aluminum, cadmium, and fluoride, and the natural aging process linked to amyloid β‐induced aging. Neurotoxic injuries, including ischemia–reperfusion and stroke (e.g., middle cerebral artery occlusion), have shown mitigation by BBR in both animal and cell‐based studies (Shaker et al. 2021; Zhu et al. 2018; Jiang, Li, and Li 2015; Ye et al. 2009; Abdel Moneim 2015; Hussien et al. 2018). Furthermore, significant advancements in nanotechnology and the adoption of nose‐to‐brain drug delivery methods have substantially improved BBR's capacity to effectively cross the BBB and accurately dispense medication to the brain (Saleh et al. 2021; Raju et al. 2021; Long et al. 2020; Tavakkoli et al. 2019). For instance, when chitosan is used to coat nanostructured lipid carriers containing BBR, these BBR‐CTS‐NLCs demonstrate enhanced brain targeting and increased effectiveness in treating neurological disorders like AD when administered intranasally (Abo El‐Enin et al. 2022).

BBR's neuroprotective mechanisms are complex and context‐dependent. Scientists have, within the last 30 years, identified multiple pathways through which BBR protects the brain, including:

- Antioxidation: BBR acts as a potent antioxidant, neutralizing reactive oxygen species (ROS) and enhancing endogenous antioxidant enzyme activities (e.g., SOD, CAT, GPx, NQO1), thereby mitigating cellular damage caused by oxidative imbalance (Shaker et al. 2021; Song et al. 2020; Zhu et al. 2018; Jiang, Li, and Li 2015; Ye et al. 2009; Abdel Moneim 2015; Hussien et al. 2018; Cheng et al. 2022; Fan, Liu, et al. 2019; Shi et al. 2020).
- Mitochondrial function improvement: BBR helps maintain mitochondrial integrity and function, which is critical for neuronal energy production and survival, particularly under stress conditions (Shaker et al. 2021; Song et al. 2020; Zhu et al. 2018; Jiang, Li, and Li 2015; Ye et al. 2009; Abdel Moneim 2015; Hussien et al. 2018; Cheng et al. 2022; Fan, Liu, et al. 2019; Shi et al. 2020).
- Inflammation reduction: BBR exerts anti‐inflammatory effects by modulating various inflammatory mediators and signaling pathways, such as inhibiting NF‐κB activation and decreasing pro‐inflammatory cytokine (e.g., IL‐1β, TNFα, IL‐6) production (Shaker et al. 2021; Song et al. 2020; Zhu et al. 2018; Jiang, Li, and Li 2015; Ye et al. 2009; Abdel Moneim 2015; Hussien et al. 2018; Cheng et al. 2022; Fan, Liu, et al. 2019; Shi et al. 2020).
- Regulation of cell death pathways: BBR can protect against various forms of neuronal cell death, including apoptosis (by modulating caspases, Bcl‐2, Bax, cytochrome c, p53, HIF‐1α), ferroptosis, and necroptosis, promoting cell survival (Shaker et al. 2021; Song et al. 2020; Zhu et al. 2018; Jiang, Li, and Li 2015; Ye et al. 2009; Abdel Moneim 2015; Hussien et al. 2018; Cheng et al. 2022; Fan, Liu, et al. 2019; Shi et al. 2020).
- Autophagy induction: BBR triggers autophagy, a vital cellular process that aids in eliminating impaired cells, aggregated proteins, and dysfunctional organelles, crucial for neuronal health and preventing the accumulation of toxic substances (Shaker et al. 2021; Song et al. 2020; Zhu et al. 2018; Jiang, Li, and Li 2015; Ye et al. 2009; Abdel Moneim 2015; Hussien et al. 2018; Cheng et al. 2022; Fan, Liu, et al. 2019; Shi et al. 2020).
- Modulation of key signaling pathways: BBR interacts with numerous crucial signaling pathways involved in cell survival, inflammation, and metabolism. These include the phosphoinositide 3‐kinase (PI3K)/protein kinase B (Akt), mitogen‐activated protein kinases (MAPKs), AMP‐activated protein kinase (AMPK), hypoxia‐inducible factor‐1 (HIF‐1), NF‐κB, peroxisome proliferator‐activated receptors (PPARs), cyclic AMP response element (CRE)‐binding protein (CREB), Nrf2, and p53 pathways (Shaker et al. 2021; Song et al. 2020; Zhu et al. 2018; Jiang, Li, and Li 2015; Ye et al. 2009; Abdel Moneim 2015; Hussien et al. 2018; Cheng et al. 2022; Fan, Liu, et al. 2019; Shi et al. 2020).
- Gut microbiota regulation: Recent studies demonstrate that oral BBR can regulate gut microbiota composition and function, which in turn influence brain dopamine levels, suggesting an intricate gut‐brain axis mechanism (Wang, Tong, et al. 2021).

## 4 BBR and AD

**FIGURE 3:**
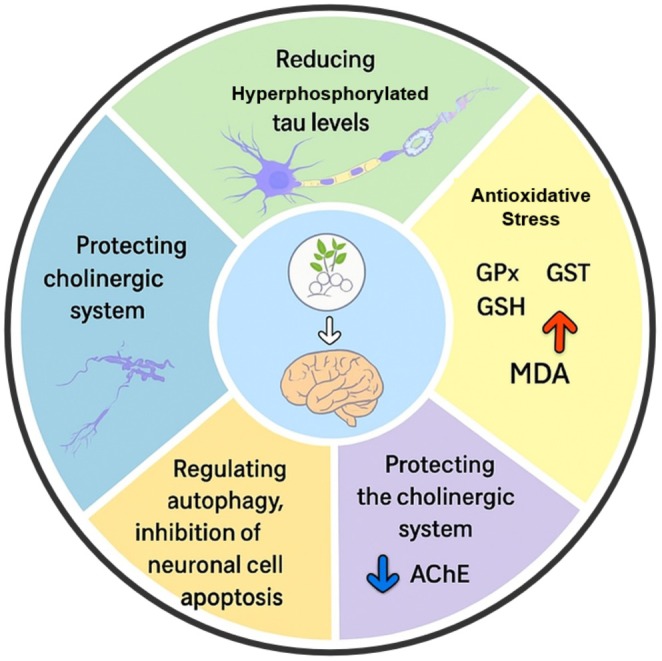
Possible mechanism of berberine on AD.

The development of AD is mainly characterized by the buildup of Aβ, excessive activation of tau protein phosphorylation, decline in cholinergic processes, neuroinflammation, heightened oxidative stress, and disruption of neuronal synaptic activity (Yin et al. 2016). BBR may protect against AD through a variety of molecular mechanisms, and further research into these could establish a stronger theoretical basis for its use in clinical settings (Figure 3). A key characteristic distinguishing AD is the build‐up of amyloid beta (Aβ) peptide, caused by anomalous breakdown of amyloid precursor protein (APP). Extensive research reveals that the sequential process of APP cleavage, resulting in Aβ production, is a critical mechanism in disease progression (Selivanova et al. 2007; Beel et al. 2010). APP is abundant in the brain and various tissues, primarily located in neuronal synapses, and is rapidly broken down through complex enzymatic processes involving β‐secretase, the γ‐secretase complex, and α‐secretase. APP breakdown occurs through two distinct pathways: the amyloidogenic pathway and the non‐amyloidogenic pathway (Deng et al. 2013; Torres et al. 2012). Amyloidogenesis is characterized by APP breakdown via β‐secretase, creating sAPPβ and C99, which is further cleaved by γ‐secretase to generate the APP intracellular domain and Aβ. The non‐amyloidogenic pathway, conversely, involves α‐secretase breaking down APP, producing sAPP alpha and a specific end portion, which is then cleaved by γ‐secretase, resulting in the release of the p3 molecule and the APP intracellular segment (Shimojo et al. 2008; Shoji et al. 1996).

**FIGURE 4:**
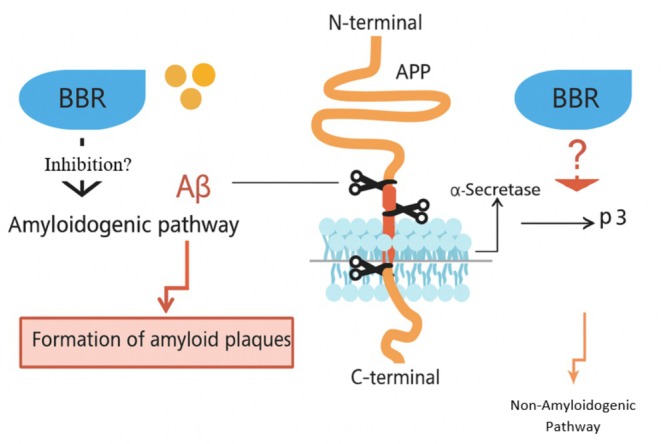
Possible mechanisms by which berberine modifies metabolism of APP.

Recent research suggests that BBR's neurological impacts may aid in improving AD symptoms by reducing Aβ production (Ji and Shen 2011; Haghani et al. 2015) (Figure 4). In a controlled laboratory study, BBR prevented Aβ‐induced production of interleukin‐6 and monocyte chemotactic protein‐1. Additionally, it inhibited the expression of cyclo‐oxygenase‐2 and induced nitric oxide synthase by blocking the mitogen‐activated protein kinase and phosphoinositide 3‐kinase/protein kinase B signaling pathways in primary microglial and BV2 cells (Jia et al. 2012). Durairajan et al. definitively demonstrated that BBR is highly successful in enhancing Aβ pathology, decreasing gliosis, and alleviating cognitive impairments in a transgenic mouse model of AD (Durairajan et al. 2012). Using BBR protected the hippocampus against neurodegeneration and ameliorated behavioral disturbances in rabbits with AD. Additionally, the treatment resulted in a decrease in BACE‐1 activity (Panahi et al. 2013). BBR's medicinal qualities have shown positive effects in several neurological disorders, including Huntington's, Alzheimer's, and Parkinson's diseases (Kumar et al. 2015).

Consistent with its general antioxidant properties (as described previously), extensive studies confirm BBR's advantageous impact on neurodegenerative disorders (Ahmed et al. 2015). Numerous pieces of evidence suggest that BBR's potent antioxidant characteristics are key to its effectiveness in shielding against AD development (Huang et al. 2016; Luo et al. 2012). Research by Luo and colleagues revealed that BBR significantly safeguards rat cortical neurons from Aβ‐induced cell death by reducing malondialdehyde (MDA) and ROS levels (Luo et al. 2012). As oxidative stress and neuroinflammation continuously influence each other, BBR can decelerate oxidative stress by impeding the advancement of neuroinflammation in the brain tissue of those with AD. Considering the harmful cycle caused by the accumulation of intracellular Aβ and the formation of NFTs, which result in oxidative damage and inflammation, BBR has the potential to decrease these pathological processes and impede the progression of oxidative stress. However, knowledge about BBR's precise impact on oxidative stress in AD is still limited, necessitating further research to gain a comprehensive understanding of its specific role.

Many scientific investigations link inflammation to AD development, though the exact mechanism by which inflammation influences disease progression remains uncertain (Kizilarslanoğlu et al. 2015; De Felice and Ferreira 2014). Neuroinflammation, a concept proposed in the 1980s and supported by retrospective data and animal studies, implies that ongoing inflammation in AD can aggravate disease advancement through mechanisms such as cytokine generation (De Felice and Ferreira 2014; Wyss‐Coray and Rogers 2012). Extensive studies demonstrate that BBR could potentially prevent AD onset by impeding this harmful cycle of neuroinflammation (Jiang, Li, and Li 2015; Jia et al. 2012; Zhu and Qian 2006).

Diabetic brain, specifically insulin resistance (IR), leads to the production of harmful Aβ and causes damage to brain axons, characteristic signs of AD in individuals with diabetes. While the exact biological process remains unclear, chronic high glucose and insulin (HGHI) exposure accelerates IR incidence in type II diabetes models. Evidence suggests that BBR could be highly effective in addressing IR both in vivo and in vitro. A recent investigation discovered that BBR protects against HGHI‐induced IR's negative effects and elucidated its precise mechanism of action. To examine the treatment of hyperinsulinemia in an insulin‐resistant model, cells were induced with HGHI, and BBR was utilized. Various techniques, including CCK8 assay, ELISA assay, Morris water maze, PET imaging, glucose kits, western blot analysis, and microscopy, assessed BBR's beneficial impacts on HGHI‐induced cells and uncovered underlying mechanisms. The beneficial effects of BBR on HGHI‐induced IR were directly linked to enhanced glucose uptake in neurons. According to the HGHI model, quantities of Pi3K, GLUT3, PKC epsilon, and APP were decreased, while p‐IRS Ser307 levels were elevated compared to the Normal group. However, BBR treatment successfully corrected these deviations. Furthermore, BBR effectively restored GSK3 beta Y216 levels, inhibited Aβ oligomer formation, and stimulated axon elongation in neurons. BBR alone positively impacts IR by correcting dysfunction of various proteins within the insulin pathway, ultimately leading to improved glucose utilization. Moreover, it effectively inhibits Aβ production and improves neuronal axon integrity. As a result, BBR proves to be an effective treatment for alleviating cognitive impairment caused by diabetes mellitus (Wu et al. 2021).

**FIGURE 5:**
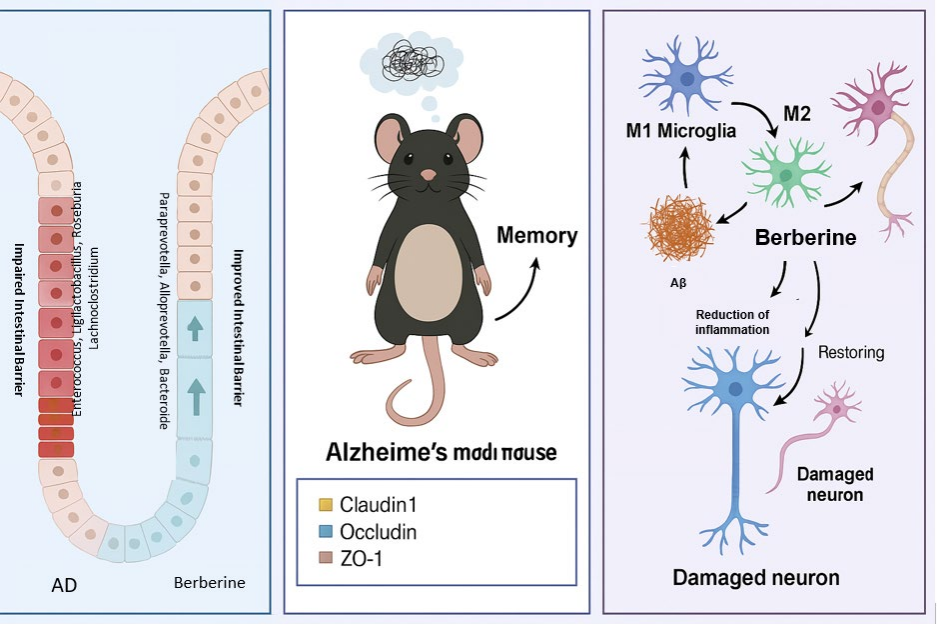
Possible effects of berberine on gut microbiota in AD.

AD contributes to a decline in mental and physical functioning, influenced by the intestinal microbiome. With its ability to decrease Aβ buildup, BBR safeguards the central nervous system. Moreover, it could potentially benefit individuals with AD by reducing excessive hyperphosphorylation of Tau and facilitating the elimination of damaged Tau proteins via autophagy. However, BBR's impact on the gut microbiome in relation to AD has not been extensively studied. A recent experiment revealed that administering BBR to 5xFAD mice ameliorated Aβ buildup in the brain, restored neurons, and decreased activation of astrocytes and microglia (Luo et al. 2012). By administering BBR, the 5xFAD mice displayed a reduction in learning and memory deficits. Analysis of mouse intestinal tissue and 16S rRNA sequences found in mouse feces showed that BBR administration reduced inflammation in the intestine, enhanced intestinal permeability, and positively changed the makeup of intestinal microorganisms and their byproducts. This ultimately helped regulate oxidative stress in the 5xFAD model. A more in‐depth examination of the genetic makeup and expression of the mouse brain showed that BBR may work to improve symptoms of neurological disorders by targeting specific molecular pathways (Figure 5) (Sun et al. 2024).

## 5 BBR and PD

Elevated levels of oxidation combined with decreased quantities of protective antioxidant enzymes ultimately result in programmed cell death (apoptosis) (Annunziato et al. 2003). Apoptosis involves two distinct routes: intrinsic and extrinsic pathways (Fulda and Debatin 2006). Caspases, a group of cysteine proteases, are recognized as indicators of apoptosis (Weng et al. 2002). Apoptosis is an essential factor in PD advancement. For example, 6‐OHDA reduces Bcl‐2 and elevates Bax, initiating apoptosis (Cao et al. 2013). Activation of caspases by 1‐methyl‐4‐phenyl‐1,2,3,6‐tetrahydropyridine (MPTP) occurs through cytochrome c release, ultimately resulting in dopaminergic cell death (Viswanath et al. 2001). Rotenone shares a comparable impact by stimulating caspases, leading to dopaminergic neurodegeneration through apoptosis (Ahmadi et al. 2003). Excessive production or alteration of α‐synuclein and amplification of mutated LRRK2 are additional factors that lead to apoptosis of dopaminergic neurons (Saha et al. 2000; MacLeod et al. 2006). Consistent with its general anti‐apoptotic properties (see “BBR and neurological disorders” section), BBR is effective in lowering apoptosis‐inducing proteins (p53, HIF‐1α) in experimental ischemia models. Moreover, it enhances defensive mechanisms against cell death by promoting Bcl‐2 synthesis and suppressing harmful proteins including caspase, Bax, and cytochrome c in motor neuron‐like cell types (Zhang, Qian, et al. 2012; Hsu et al. 2012). Studies on ischemia models demonstrate that BBR effectively hinders neuronal apoptosis by activating the PI3K/Akt pathway. Kim et al. explored BBR's effects on short‐term memory in a PD mouse model by observing changes in dopamine (DA) levels and neurogenesis in the hippocampus. Oral BBR for 5 weeks improved memory by preventing hippocampal cell death in PD mice, leading to improved motor function and less degeneration of DA‐producing neurons (Kim et al. 2015).

Moreover, research demonstrates that BBR can substantially reduce the harmful effects of oxidative stress (Kahale et al. 2014). Bae and colleagues investigated different processes triggered by BBR to prevent neuronal cell death in PD. Their research showed that BBR considerably reduces ROS levels, inhibits caspase‐3 activation, and protects SH‐SY5Y cells from 6‐OHDA‐induced death (Bae et al. 2013). Thus, it is likely that BBR's ability to inhibit caspase‐3 is responsible for its anti‐apoptotic effects in PD.

The characteristic features of PD include a decrease in DA levels and dysfunction in the cholinergic system (Berendse et al. 2001; Harnois and Di Paolo 1990; Höglinger et al. 2004; Aarsland et al. 2004; Müller and Bohnen 2013). Tyrosine hydroxylase (TH) is a crucial enzyme for DA production, controlling its synthesis rate. TH requires the coenzyme BH4, which acts as an electron transport agent, aiding in the tyrosine conversion to L‐DOPA (DA precursor), and also serves as a reducing agent, stabilizing TH's active form and improving its efficiency (Cumming and Gjedde 1998). BH4 absence inhibits Phe to Tyr transformation and Tyr to dopa conversion, disrupting the Phe‐Tyr‐dopa‐DA metabolic pathway (Ishikawa et al. 2016). Recent animal investigations yield conflicting findings on BBR's impact in PD models. In one study, low doses (25–100 mg/kg) of BBR were linked to decreased ChE levels in the striatum, cortex, and hippocampus of 6‐OHDA‐induced PD rats, as well as reduced dopaminergic neuron degeneration in MPTP/P‐induced PD mice. Additionally, it increased TH‐positive neurons and decreased DA, DOPAC, and HVA levels in 6‐OHDA‐induced PD rats (Kim et al. 2015; Kahale et al. 2014; Negahdar et al. 2015; Shin et al. 2013). Conversely, another research shows opposing results in 6‐OHDA‐induced PD rats, indicating that BBR does not positively impact DA‐producing neurons in 6‐OHDA‐induced lesions and may even contribute to increased neuronal damage when 6‐OHDA is administered (Kwon et al. 2010).

lncRNAs participate in PD development. One particular lncRNA, LINC00943, is elevated in PD. Silencing LINC00943 reduced neuronal damage in a PD experimental model by regulating miR‐7‐5p/CXCL12 and miR‐15b‐5p/RAB3IP pathways (Lian et al. 2021; Meng et al. 2021). Furthermore, LINC00943 reduction played a crucial role in inhibiting PD advancement through miR‐338‐3p/SP1 pathway regulation (Sun et al. 2022). New research shows that BBR effectively blocked cell death, oxidative damage, and inflammation in PD by specifically targeting the LINC00943/miR‐142‐5p/KPNA4/NF‐kappaB pathway (Li et al. 2021). Exploring the connection between BBR and lncRNAs may offer valuable information on how to effectively manage PD.

It is evident that there is a close relationship between PD and gut dysbiosis. The intestinal tract of individuals with PD presents with inflammation and decreased levels of SCFAs in their stool (Vascellari et al. 2020). These changes are linked to gastrointestinal dysbiosis (Aho et al. 2021). When BBR is ingested orally, it notably influences gut microorganism makeup. Additionally, BBR has the capability to improve gut bacteria performance, leading to SCFA creation, which has various positive health effects (Wang, Guo, et al. 2017). Another significant impact of BBR is its ability to increase butyrate production by gut bacteria. This substance is then transported into the blood and has been proven to successfully reduce both blood sugar and fat levels (Wang, Shou, et al. 2017). BBR significantly raised Tyr, dopa, and DA levels in the gut flora, while Phe showed a decrease. This is because BBR aids in BH4 production by providing H•, which in turn boosts TH activity. In addition, oral BBR significantly improved dopa/DA production by gut microorganisms. As a result, dopa/DA levels were raised in the blood and also in the brain. Hence, the connection between BBR and gut microbiota could potentially offer a novel approach to addressing PD (Wang, Tong, et al. 2021).

## 6 BBR and MS

As the nervous system deteriorates over time, MS is characterized by numerous lesions that damage the myelin sheath in both the spinal cord and brain (Manu et al. 2021; Zahid et al. 2021). BBR has shown promise as a viable treatment method for managing MS symptoms. In an experimental autoimmune encephalomyelitis (EAE) model, BBR has been noted to reduce BBB permeability and disrupt MMP‐9 activity in the cerebrospinal fluid and brain of both individuals and animals suffering from the disease (Ma et al. 2010). Additionally, BBR is capable of suppressing gelatinase function and decreasing laminin damage (Jiang et al. 2013). Simply put, the more severe MS becomes, the worse reactive gliosis becomes. It is uncertain if BBR, known for its anti‐inflammatory properties (as discussed generally), can effectively treat MS; further research is needed to determine its effectiveness (Luo et al. 2017).

BBR was observed to have a stronger effect on mature DCs compared to immature DCs when stimulated with LPS. Consequently, there was a decline in CD80/CD86 and IL‐12 expression when BBR was present during LPS stimulation (Hu et al. 2011). BBR has been observed to have various effects on mature dendritic cells activated by LPS. These effects include inhibition of costimulatory molecule production (CD40, CD86, CD80) and increased IL‐23 production, while simultaneously decreasing IL‐6 and IL‐1βlevels (Yang et al. 2013). Moreover, BBR can induce apoptosis in DCs (Karimi et al. 2017). It is essential to emphasize the necessity of conducting more studies to gain a comprehensive understanding of BBR's effects on DC function and the mechanisms responsible for it.

**FIGURE 6:**
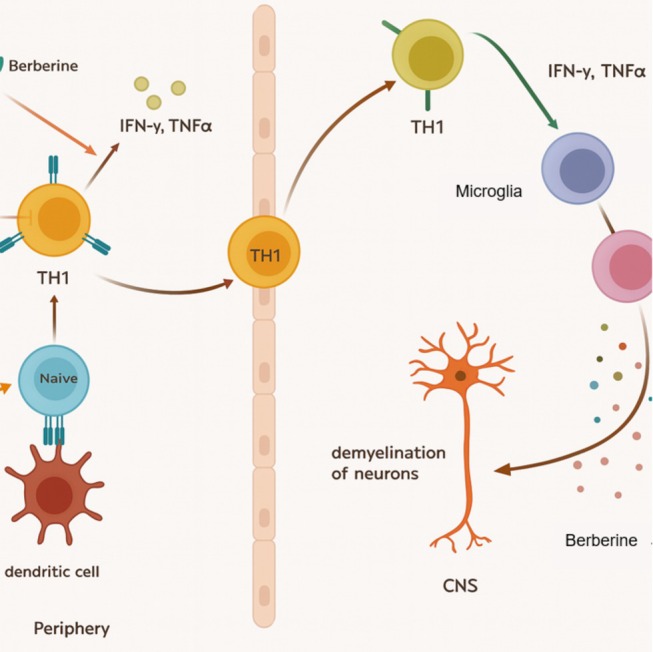
Naive T cells develop into Th1 cells by displaying antigens and releasing cytokines, especially IL‐12, with the assistance of dendritic cells. These Th1 cells can enter the BBB and avoid being controlled by the immune system. This results in the activation of microglia, which are macrophages found in the CNS, due to the release of cytokines like IFN‐γ. These microglia then convert into M1 cells and can cause damage to neurons by producing ROS and inflammatory cytokines. Berberine's ability to regulate IL‐12 leads to a reduction in the expression of STAT4, STAT1, and T‐bet in immature T cells, ultimately leading to a decrease in the number of Th1 cells. Furthermore, it has the potential to lower the production of inflammatory cytokines from both Th1 cells and M1‐macrophages, and mitigate the harmful effects of M1‐produced ROS.

Existing evidence hints at the possibility of BBR hindering the conversion of naïve CD4+ T cells to Th1 by decreasing the activation of STAT1 and STAT4, which are triggered by IL‐12 signaling. Furthermore, BBR may also curb T‐bet production (Tavaf et al. 2023; Liu et al. 2016; Qin et al. 2010; Tong et al. 2016). In experiments with EAE mice, BBR administration led to a reduction in the Th1 cell population by inhibiting the impact of IL‐12 signaling on unactivated T cells (Figure 6) (Qin et al. 2010). Using the experimental autoimmune neuritis model, commonly used for studying autoimmune disorders of peripheral demyelination such as Guillain–Barre syndrome, it has been demonstrated that BBR can suppress Th1 cytokine synthesis, particularly TNF‐α, ultimately decreasing symptom intensity (Li et al. 2014). After conducting additional investigation, it was revealed that BBR has the ability to stimulate IL‐12 production, thereby promoting Th1 differentiation (Kang et al. 2002). There is a belief that BBR's effects can ultimately alleviate conditions such as asthma, where the Th2 pathway is a significant factor (Kips et al. 1996).

BBR can be a valuable treatment for autoimmune diseases as it plays a crucial role in countering Th17 responses, which contribute remarkably to the development of these conditions. Hence, exploring BBR's impact on these cells can provide significant insights into the effective management of autoimmune diseases. During an EAE experiment, BBR was found to block the progression and activity of Th17 cells by directly impacting the JAK/STAT signaling pathway. As a consequence, there is a reduction in ROR‐gammat levels and STAT3 activation, leading to the obstacle in Th17 cell differentiation. Additionally, BBR has the potential to improve the state of EAE by indirectly affecting Th17 cells through alterations in gut microbiota. This is accomplished by restraining NF‐kB activity in CD11b + APCs, which leads to reduced levels of costimulatory molecules and IL‐6 cytokine production. Consequently, BBR has a beneficial effect on EAE (Qin et al. 2010). A recent investigation provided further evidence that BBR effectively hindered the production and differentiation of Th17 cells by directly suppressing IL‐17 and indirectly affecting DCs in individuals with Vogt‐Koyanagi‐Harada disease (Yang et al. 2013). The administration of BBR to NOD mice with type 1 diabetes resulted in the prevention of Th17 differentiation. The successful inhibition of Th17 differentiation was accomplished by activating ERK1/2, suggesting the possibility of ERK's suppressive function in this process. By suppressing the STAT3 and ROR‐γt signaling pathways, ERK effectively hinders Th17 differentiation, suggesting the potential use of ERK as a target for treating diseases related to Th17 activity (Noack and Miossec 2014). According to the research conducted by Mengfan Yue et al. oral BBR has shown promising results in reducing collagen‐induced arthritis in rats, which mimics human rheumatoid arthritis. This positive effect can be credited to the inhibition of Th17 cell reactions, linked to higher cortistatin (a neuropeptide that regulates the immune system) production in the gut. Cortistatin mRNA and protein levels were significantly increased in the intestines of BBR‐treated rats, resulting in a reduction of Th17 cell activity throughout the body (Yue et al. 2017). Recent studies indicate that BBR can effectively lessen overactive Th17 cell responses and lower Th17 cytokine levels in a rat model of experimental autoimmune myocarditis (EAM), thus enhancing EAM condition. This therapeutic property of BBR on Th17 cells can be attributed to its ability to prohibit STAT3 signaling pathway activation (Liu et al. 2016). A different experiment on EAE mice found that BBR inhibits the transformation of untreated CD4+ T cells into harmful Th17 cells by directly affecting the JAK/STAT pathway. This leads to reduced inflammation and improvement in the condition of EAE‐affected mice (Qin et al. 2010). Research on EAE mice conclusively proves that BBR treatment effectively decreases the presence of key transcription factors (ROR‐γt) and inflammatory cytokines (such as IL‐17) produced by Th17 cells. This reduction in inflammatory markers correlates with a decrease in central nervous system inflammation and damage to the myelin (Tavaf et al. 2023). The findings suggest that BBR may positively impact reducing destructive Th17 cell activation in EAE progression. Moreover, it exhibits promise in decreasing inflammation and fostering a more favorable immune reaction in MS patients.

## 7 BBR on Stroke

A stroke is one of the main factors leading to death and prolonged disability, occurring when there is insufficient circulation to a particular part of the brain, or when there is bleeding in the brain tissue or the adjacent subarachnoid space (Chugh 2019). To prevent or treat stroke, various measures can be taken, such as recanalization, thrombolysis, primary prevention, secondary prevention, neuroprotection, and neurorepair (Kuriakose and Xiao 2020; Kinlay 2011). In both pre‐treatment and post‐treatment scenarios, BBR has demonstrated significant effectiveness in treating stroke (Figure 6). Studies reveal that BBR acts as a thrombin inhibitor and can prevent thrombin‐induced platelet aggregation in washed platelet samples when tested in a laboratory setting. However, there is currently no research on BBR's potential thrombolytic effects in living organisms (Wang, Zhang, et al. 2017).

The MCAO procedure is frequently utilized to create a valid mouse model of stroke (Liu and McCullough 2011). Once cerebral infarction takes place, the release of oxidative components and pro‐inflammatory cytokines ensues, resulting in neuronal death due to ischemia, which involves both apoptosis and necrosis (Manzanero et al. 2013). After experiencing a lack of oxygen and then being reintroduced to it, a series of inflammatory reactions begins. The HMGB1 protein is released from dead nerve cells, which then activates the NF‐κB pathway, a common measure of inflammation in stroke studies (Zhu et al. 2018; Majid 2014). Subsequently, IL‐1β, TNF‐α, and IL‐6 are activated (Ramiro et al. 2018; Yang et al. 2019). In a span of 7 days, BBR successfully impeded NF‐κB translocation to the nucleus and obstructed pro‐inflammatory cytokine generation. Consequently, there was a reduced level of inflammatory compounds such as IL‐1β, TNF‐α, and IL‐6, while also promoting anti‐inflammatory cytokine secretion like IL‐10 (Zhang, Fu, et al. 2017).

As previously discussed, oxidative stress is a significant factor in stroke (Pradeep et al. 2012), where an overabundance of ROS leads to neuronal harm and demise (Majid 2014). Consistent with its antioxidant properties, BBR pre‐treatment led to a reduction in elevated MDA levels and a positive impact on antioxidative enzyme performance, including SOD, CAT, peroxiredoxin, and NQO1 (Zhang, Fu, et al. 2017; Chen et al. 2016). Moreover, prior BBR administration effectively decreased neural cell death by inhibiting caspase enzymes (caspase‐9 and caspase‐3) and upregulating Bcl‐2 expression. Furthermore, BBR activated cell‐survival mechanisms such as AKT phosphorylation and increased ERK1/2 levels (Zhang, Qian, et al. 2012; Hu et al. 2012; Simões Pires et al. 2014; Yang et al. 2018). BBR, by attaching to the poly (A) tail of retinoblastoma mRNA, counteracts mRNA degradation and enhances retinoblastoma protein expression in ischemia/reperfusion cases. As a result, apoptosis was hindered, and cell survival was promoted in the damaged brain (Chai et al. 2014).

**FIGURE 7:**
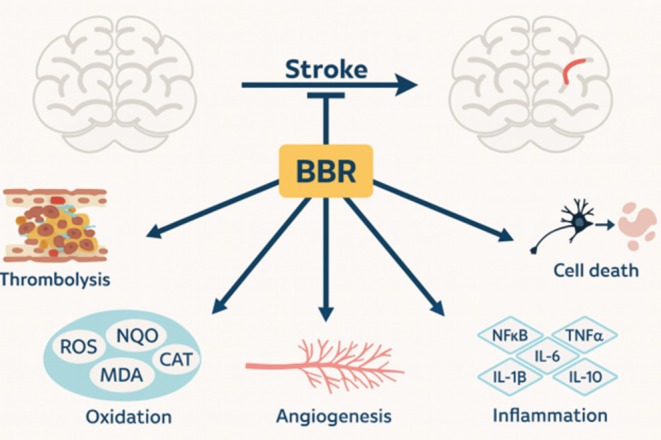
Effects of BBR against stroke.

Furthermore, administering BBR post‐MCAO surgery produced comparable results to pre‐surgery treatment in reducing infarction volume in both rats and mice (Maleki et al. 2018; Zhang, Zhang, et al. 2012; Shou et al. 2022). BBR exhibited potent abilities to decrease damage caused by focal cerebral ischemia by upregulating IL‐10 levels and inhibiting NF‐κB movement to the nucleus. It also facilitated an increase in claudin‐5 expression to reinforce the protective function of the BBB (Maleki et al. 2018; Zhang, Zhang, et al. 2012). The reduction of harmful ROS was a crucial element in the positive outcomes observed after administering BBR. Our investigations demonstrate that BBR functions as a potent stimulator of peroxisome proliferator‐activated receptor delta (PPARẟ), resulting in elevated levels of nuclear factor (erythroid‐derived 2)‐like 1/2 (NRF1/2) and NQO1. This mechanism effectively decreases ROS in the brains of MCAO‐afflicted mice, highlighting BBR's capacity to safeguard against neuronal damage and facilitate post‐stroke healing through its ROS‐regulating abilities (Shou et al. 2022). In addition, BBR has the advantage of promoting angiogenesis development through the regulation of AMPK‐dependent M2 macrophage/microglial polarization, playing a crucial role in enhancing functional recovery from ischemic stroke (Zhu et al. 2019). Overall, BBR is highly effective in reducing stroke damage, mainly by promoting blood clot breakdown, reducing oxidative and inflammatory damage, preventing nerve cell death, and promoting angiogenesis (Zhang, Qian, et al. 2012; Wang, Zhang, et al. 2017; Zhang, Fu, et al. 2017; Chen et al. 2016; Hu et al. 2012; Simões Pires et al. 2014; Yang et al. 2018; Chai et al. 2014; Zhang, Zhang, et al. 2012; Shou et al. 2022; Zhu et al. 2019) (Figure 7).

## 8 BBR and SCI

The use of BBR can protect neurons in both neuroinflammation and neurodegenerative diseases. However, its effects on acute SCI are still not fully understood and require further investigation. Wang et al. assessed BBR's ability to protect the nervous system in SCI instances. They utilized Sprague–Dawley rats to simulate SCI and then administered BBR via abdominal injection after the injury. The scientists performed a comprehensive evaluation of neurological functioning, analyzing levels of pro‐inflammatory indicators like TNF‐α and IL‐1$\beta$, as well as proteins associated with autophagy (LC3B, ATG7, ATG16L), and an apoptosis‐related protein, cleaved caspase‐3. Furthermore, they observed CNPase, a specific marker for oligodendrocytes, and autophagy‐related proteins ATG5 while investigating ventral horn neurons. In a controlled laboratory environment, levels of pro‐inflammatory factors TNF‐α and IL‐1β were measured in primary spinal neurons exposed to lipopolysaccharide. BBR demonstrated the ability to decrease LPS‐triggered inflammatory signals in primary spinal nerve cells. Nonetheless, this effect was reversed when the autophagy inhibitor 3‐Methyladenine was present. These findings suggest that BBR can hinder inflammatory reactions and cell death in the injured spinal cord by promoting autophagy in oligodendrocytes, ultimately aiding in nerve cell regeneration (Wang, Liu, et al. 2017).

In recent times, scientists have shown great interest in Exosomes (Exos). These small structures are essential for cell‐to‐cell communication, transporting various molecules, including proteins, mRNAs, and miRNAs through paracrine secretion (Hannafon and Ding 2013; Ludwig and Giebel 2012).

Inheriting the cell membrane from their parental cells, Exos exhibit excellent biocompatibility and evade immune clearance, leading to prolonged circulation within the body (Lin, Lu, and Li 2020). Moreover, Exos possess the capability to reduce inflammation and successfully cross the BBB, a well‐documented phenomenon (Wang, Tang, et al. 2021). Hence, Exos are a highly suitable method of delivering medication for SCI treatment. These microscopic vesicles, produced by mononuclear macrophages, regulate inflammation levels. Furthermore, macrophage‐derived Exos possess potent anti‐inflammatory and neuroprotective properties, making them effective in countering SCI effects (Zhang, Li, et al. 2021). After SCI, microglia, a specific type of immune cell in the brain and spinal cord, become stimulated and produce substances that draw peripheral macrophages to the affected area. These activated macrophages and microglia share the M1 subtype, characterized by its role in causing inflammation and harm to nerve cells (Zhou et al. 2014; Nakajima et al. 2020). Conversely, the M2 subtype of activated macrophages/microglia possesses significant anti‐inflammatory and neuroprotective abilities (Xu et al. 2018).

In their scientific investigation, Gao and their team created exosomes from primary peritoneal macrophages of the M2 variety to serve as transporters for BBR. By harnessing exosomes' distinct capability to penetrate the BBB, this approach successfully transports medication to the damaged spinal cord tissue. The researchers successfully loaded Exos with particles measuring 125 ± 12 nm through ultrasonic techniques, resulting in a drug loading rate of 17.13% ± 1.64%. The drug release experiment showed a sustained release pattern, with 71.44% ± 2.86% of the drug released within 48 h. In vitro and in vivo experiments demonstrated that the Exos‐Ber mixture successfully decreased the M1 protein iNOS and encouraged an increase in the M2 protein CD206. Moreover, in animal studies, it was observed to lower inflammatory and apoptosis signaling molecules, including IL‐1beta, TNF‐α, Caspase 9, Caspase 8, and IL‐6. The findings suggest that Exos‐Ber can significantly decrease cell death and inflammation by stimulating M1 macrophage/microglia transformation to M2. Moreover, it has demonstrated encouraging results in enhancing motor abilities in mice with SCI, highlighting its potential as a therapeutic option for this disorder. The targeting strategy for drug delivery utilizing M2 macrophage‐derived exosomes has also been found effective due to their biocompatibility, stealth abilities, and natural ability to target inflammation. After SCI, the body's inflammatory environment strongly supports using exosomes for targeted treatment. Exosomes, being natural drug carriers, offer a higher level of safety. While nanomaterials have greatly advanced drug carrier selectivity, the specific microenvironment after a central nervous system injury can cause certain inorganic materials to increase pressure and potentially cause secondary neuronal damage. Fortunately, exosome introduction has solved this issue. While some scientists have previously used exosomes from tumor cells as drug carriers, the danger of carrying carcinogenic factors makes this method extremely risky. In comparison, using exosomes from endogenous macrophages offers a much safer alternative (Gao et al. 2021).

## 9 BBR and Other Neurological Disorders

Huntington's disease (HD) is a hereditary condition primarily manifesting as chorea, dystonia, impaired motor skills, and cognitive decline (McColgan and Tabrizi 2018). HD occurs due to an unusually high amount of CAG repeats within the huntingtin gene, leading to an extended polyglutamine sequence in the huntingtin protein (Ross and Tabrizi 2011). BBR significantly enhanced motor capabilities and extended the lifespan of N171‐82Q HD mice by boosting autophagy (as generally described) and diminishing mutant huntingtin accumulation (Jiang, Wei, et al. 2015).

A traumatic brain injury (TBI) is a condition in which the brain is harmed by an external force, leading to potential bruising, torn tissues, bleeding, and other forms of physical harm. This type of injury can result in lasting issues or even fatalities (Galgano et al. 2017). TBI results in neurological dysfunction caused by both direct and indirect damage. The initial damage is responsible for the main injury; however, the subsequent harm is caused by the pathological alterations that ensue (Werner and Engelhard 2007). A significant factor that can impact TBI recovery is secondary injury. However, BBR treatment has demonstrated promising results in reducing secondary injury. Research demonstrates that BBR has the capability to reduce cortical lesion size and prevent neuronal loss by hindering microglia and astrocyte activation in both the cortical lesion border zone and the hippocampal CA1 region. Furthermore, BBR is effective in lowering oxidative and inflammatory harm (consistent with its known mechanisms) by suppressing iNOS and COX‐2 enzyme expression. These functions contribute to BBR's effectiveness as a treatment after initial medical intervention (Huang et al. 2018). Studies also discovered that BBR treatment following an injury blocked TLR4/MyD88/NF‐kappaB signaling pathway activity. As a result, there was a reduction in the inflammatory response in glial cells, ultimately resulting in better outcomes for TBI (Chen et al. 2014). In summary, BBR's preventative and curative effects against a multitude of illnesses can be attributed to its ability to act as an antioxidant and reduce inflammation (Huang et al. 2018; Chen et al. 2014).

Dementia is characterized by a collection of indications that affect memory loss and mental functioning. While not a distinct illness, research shows that brain disorders and the natural aging process can lead to dementia development (Wimo et al. 2017; Wahl et al. 2019). AD is the primary contributor to dementia cases, accounting for around 60%–70% of all dementia cases worldwide (Wimo et al. 2017). Approximately 20% of dementia cases are attributed to vascular dementia, making it the second most prevalent form. BBR is mainly used to target symptoms related to dementia caused by AD. Furthermore, research demonstrates its positive effects in treating vascular dementia, a disorder typically stemming from reduced blood circulation to the brain (Iadecola 2013). BBR treatment in the vascular dementia model, triggered by chronic cerebral hypoperfusion, not only safeguarded against cognitive deficiencies but also inverted harm inflicted on nerve cells by chronic cerebral hypoperfusion (Aski et al. 2018). BBR showed promising results in improving blood flow in the posterior cerebral artery in a diabetes‐induced model of vascular dementia. This was achieved by inhibiting abnormal miR‐133a expression in the vascular endothelium, resulting in a notable enhancement of vascular function in the middle cerebral artery (Yin et al. 2019).

Cognitive dysfunction can be caused by harmful neurotoxic chemicals, including DOX, d‐galactose, and lipopolysaccharide. BBR use has been shown to greatly alleviate cognitive impairment in rats exposed to DOX or lipopolysaccharide, while also enhancing its antioxidant and anti‐inflammatory properties. BBR effectively decreased oxidative stress by promoting important enzyme functionality (SOD, GPx, GSH, CAT). Additionally, BBR combats inflammatory processes by inhibiting TLR4, COX‐2, NF‐κB, IL‐6, and TNF$\alpha$ expression (Shaker et al. 2021; Sadraie et al. 2019). In d‐galactose‐induced dementia rats, BBR effectively improved memory impairment by regulating Arc expression, which preserves normal synaptic plasticity, making it a key factor in memory loss amelioration (Zhan et al. 2014). Ultimately, BBR alleviates dementia symptoms by improving blood flow to the brain, minimizing harmful oxidative and inflammatory effects, and preserving healthy synaptic connections (Shaker et al. 2021; Yin et al. 2019; Sadraie et al. 2019; Zhan et al. 2014).

Schizophrenia is characterized by persistent or recurrent episodes of psychosis, with key symptoms such as distorted perceptions, abnormal thought patterns, and strange behaviors (Owen et al. 2016). Administration of the NMDA receptor antagonist, MK‐801, to rodents leads to schizophrenia‐like behaviors. Treatment with BBR improved learning deficits, although the underlying mechanism is yet to be studied (Ghotbi Ravandi et al. 2019).

Anxiety and depression have a complex, closely tied relationship, where people suffering from depression frequently also experience anxiety disorders, and vice versa. This can be attributed to shared root causes such as genetic predispositions, life events, and brain chemistry imbalances (Tiller 2013). Anxiety and depression have a complex and diverse range of causes, with various factors such as imbalanced chemical levels, environments, and genetics potentially contributing (Penninx et al. 2021). According to Figure 4, BBR proves advantageous in treating both anxiety and depression. Individuals struggling with drug dependency, specifically with substances like methamphetamine and morphine, often experience anxiety and depression symptoms. However, BBR significantly reduces these negative effects (Rezaeian et al. 2020; Lee et al. 2012; Alavijeh et al. 2019). BBR affected the central noradrenergic system in morphine‐addicted animals by ameliorating hippocampal brain‐derived neurotrophic factor (BDNF) level decrease and inhibiting locus coeruleus activation (Lee et al. 2012). BBR effectively improved methamphetamine‐induced anxiety through multiple mechanisms. It decreased neuroinflammation by reducing TLR4 and NF‐κB activity and raised oxytocin receptor levels in the nucleus accumbens and hippocampus. This led to a reduction in oxytocin levels, which has been linked to drug abuse (Rezaeian et al. 2020; Alavijeh et al. 2019; Mühlethaler et al. 1984). Moreover, medical research indicates that the menopausal transition period is associated with a heightened likelihood of developing anxiety and depression (Mulhall et al. 2018; Cyranowski 2022; Cohen et al. 2006). BBR induced a similar effect as antidepressants in ovariectomized mice, attributed to BDNF/cAMP‐response element binding protein (CREB)/eukaryotic elongation factor 2 (eEF2) pathway and 5‐HT2 receptor activation (Fan et al. 2017). Furthermore, research shows that BBR can control gut microorganism composition, leading to anxiety reduction. Specifically, studies on ovariectomized rats revealed that BBR encouraged beneficial bacteria development (Akkermansia, Bacteroides, Bifidobacterium, and Lactobacillus) in the gut. This increased equol production, which effectively improved anxiety‐related symptoms in postmenopausal individuals (Fang et al. 2021).

## 10 Challenge to New Derivatives and Formulations of BBR

Despite the fact that BBR salts containing chloride or sulphate have improved solubility and are commonly utilized in medical settings, the considerable obstacle that remains is the low bioavailability and inadequate pharmacokinetic characteristics of BBR (Fan, Zhang, et al. 2019). Development of novel compounds with a similar mechanism of action but with improved pharmacological parameters is a crucial goal. Extensive research is being conducted on BBR derivatives that would exhibit stronger biological activity at lower concentrations. Recent studies from 2019 and 2020 have shown promising results, as they have found a significant correlation between the structure of the derivatives and their activity (Wang, Deng, et al. 2020). Completely rephrased: Emerging derivatives demonstrate comparable biological parameters and show potential for additional investigation (Zhang et al. 2019). Up until now, a study has been conducted on the effects of different BBR‐12‐amine derivatives on halting the growth of human cancer cells. These derivatives include quaternary 12‐aminoberberine chloride, quaternary 12‐nitroberberine chloride, tertiary 12‐aminotetrahydroberberine, and various levels of reduced 12‐aminoberberine derivatives, all of which have shown to have the ability to inhibit the proliferation of human cancer cells:

The types of cells utilized in this research were BGC‐823 (gastric cancer), HCT‐8 (colorectal cancer), A549 (lung cancer), HeLa (cervical cancer), and Bel7402 (liver cancer). It was observed that quaternary derivatives of BBR, namely 12‐N, N‐di‐n‐alkylamine chlorides, exhibited notably greater effectiveness when compared to their reduced forms. The effects of these compounds were found to rise with an increase in the n‐alkyl carbon chain length of 12‐N, N‐di‐n‐alkylamino, within the range of about 6–8 carbon atoms. However, the activities decreased when the n‐alkyl carbon chain was extended beyond 6–8 carbon atoms. It is notable that the tertiary amine structure exhibited much higher activity than the secondary amine structure (Wang, Deng, et al. 2020).

The new versions of BBR that have been substituted with an alkyl group at the 13 positions have demonstrated superior efficacy in targeting human cancer cells compared to the original form. This was particularly evident in MDA‐MB‐231 cells, known for their radioresistance, where treatment with 13‐ethylberberine, a variation specifically designed for breast cancer, resulted in a decrease in proapoptotic gene expression and an increase in antiapoptotic gene expression compared to the original BBR compound. Moreover, the growth and ability to form clusters were hindered in both cell lines when exposed to this substance. The process behind this was related to 13‐ethylberberine, which caused cells to undergo programmed cell death by increasing the amount of ROS in the mitochondria and overall cellular surroundings. It also controlled proteins associated with the internal pathway of programmed cell death, instead of the external pathway. These derivatives have comparable efficacy and could potentially serve as medicinal remedies, in a similar manner to BBR. However, additional studies are necessary to fully understand their absorption and distribution in the body, in comparison to BBR (Jin et al. 2019).

The development and assessment of BBR derivatives that contained 9‐O‐substituted groups resulted in favorable results. Particularly, one derivative (9‐(3‐bromopropoxy)‐10‐methoxy‐5,6‐dihydro‐[1,3]dioxolo[4,5‐g]isoquino[3,2‐a]isoquinolin‐7‐ylium bromide) showed significantly improved abilities to inhibit cell growth and induce apoptosis in leukemia cells, with a 30‐fold and 6‐fold increase, respectively, when compared to BBR alone (Milata et al. 2019). Moreover, the evaluation of different forms of BBR compounds, including those with cis‐substituents at positions C9 and C13, as well as 13‐[CH2CO‐Cys‐(Bzl)‐OBzl]‐BBR and variants with a modified 9‐O position, was presented (Wang, Wang, et al. 2020; Lin, Ho, et al. 2020; Li et al. 2019; Liu et al. 2020; Du et al. 2020). The inclusion of the methylene‐dioxy and methoxyl groups has a vital impact on the anticancer effectiveness of BBR derivatives (Leyva‐Peralta et al. 2019). Currently, a study is in progress to investigate how bioactive BBR (BBR that has been mixed with Quillaja extract) is recruited and utilized by the human body.

## 11 Clinical Trials and Therapeutic Applications

The extensive findings from various clinical studies highlight the diverse uses of BBR in therapy. BBR and barberry ( *Berberis vulgaris* ) have been extensively tested in randomized clinical trials for the treatment of various illnesses, with the most researched outcomes being its ability to lower lipids and enhance insulin sensitivity. Furthermore, through clinical investigations, BBR has been explored for its potential advantages in areas such as cardiovascular health, cancer prevention, gastrointestinal disorders, neurological conditions, and endocrine disorders. Notably, the act of taking BBR by mouth has been shown to have little toxicity and hardly any negative effects when given at typical dosages, although a few individuals have experienced mild stomach‐related responses (Imenshahidi and Hosseinzadeh 2019).

A randomized clinical trial involving 55 individuals diagnosed with acute ischemic stroke found that administering a combination of BBR and 20 mg/day of atorvastatin was more successful in treating the condition compared to the use of 20 mg/day atorvastatin alone (Fei‐qi et al. 2015). Another study found that when BBR was given orally at a daily dosage of 1 g (0.5 g twice a day) for a period of 16 weeks, it led to noteworthy improvements in the composition and functioning of gut microbiota in participants. Additionally, the addition of Bifidobacterium probiotics to the regimen further increased the efficacy of BBR in lowering blood sugar levels (Ming et al. 2021). However, interpreting this solely as a direct implication for PD treatment requires caution. The study measured systemic DA, not brain DA, and was conducted in patients with hyperlipidemia, not diagnosed PD. Future clinical trials directly assessing BBR's impact on motor and non‐motor symptoms in PD patients, along with brain‐specific biomarkers (e.g., neuroimaging for striatal DA transporters), are essential to validate this hypothesis. Li et al. discovered that administering oral BBR to patients with acute cerebral ischemic stroke (AIS) effectively decreased serum IL‐6 and IMT levels. This resulted in a notable reduction in carotid atherosclerosis and an improvement in neurological function and prognosis for these patients (Li et al. 2016). However, the precise mechanism by which BBR enhances neurological function directly in AIS patients, beyond systemic effects, requires further dedicated investigation within a clinical context. According to recent research, administering 0.5 g/day of BBR orally for 8 weeks substantially increased the levels of DA in the blood of patients with hyperlipidemia. This indicates that BBR could be beneficial in the treatment of PD (Wang, Tong, et al. 2021). Extensive efforts have been dedicated toward studying the potential of BBR in treating neurological ailments in clinical trials. However, it is necessary to continue researching in this area for additional advancement.

While the preclinical data on BBR's neuroprotective effects are well‐documented and highly promising, the translation of these findings into effective human clinical therapies for age‐related neurological disorders presents several significant challenges.

Complex pathophysiology of neurological disorders: Unlike many other conditions, neurological disorders such as AD, Parkinson's disease, and ischemic stroke involve intricate and multifaceted pathogenic pathways. Preclinical models often target specific aspects of these diseases, which may not fully capture the complexity of the human condition. For instance, animal models of neurodegeneration may not perfectly mimic the slow progression and multifactorial nature of human neurodegenerative diseases, making it difficult to extrapolate efficacy (Jucker 2010; Dawson et al. 2018; Sandoe and Eggan 2013).

Dose translation and pharmacokinetics/pharmacodynamics: Determining the optimal and safe therapeutic dose of BBR in humans based on preclinical data is a considerable hurdle. Animal studies often use higher, sometimes supraphysiological, doses that may not be feasible or safe in humans. Furthermore, species‐specific differences in absorption, distribution, metabolism, and excretion (ADME) of BBR, as well as its bioavailability and ability to cross the BBB, can significantly impact its efficacy in human brains (Jucker 2010; Thomas et al. 2021; Ai et al. 2021; Vugmeyster et al. 2012; McGonigle and Ruggeri 2014). While some studies suggest BBR can cross the BBB, the extent and clinical relevance of this penetration in various neurological conditions require further investigation.

Heterogeneity of patient populations: Human populations are genetically diverse and present with varying disease severities, comorbidities, and medication regimens, all of which can influence treatment response (Macheras and Iliadis 2016; Mangoni and Jarmuzewska 2021). Preclinical studies typically use genetically uniform animal strains under controlled conditions, which simplifies data interpretation but limits generalizability to heterogeneous human populations.

Long‐term efficacy and safety: Neurological disorders often require long‐term treatment. While existing clinical trials suggest low short‐term toxicity for BBR, comprehensive long‐term safety data, especially concerning potential interactions with other medications commonly prescribed for age‐related conditions, are still accumulating. The potential for long‐term adverse effects or drug–drug interactions needs rigorous investigation in larger and longer clinical trials (Thomas et al. 2021; Dong et al. 2013; Moreira et al. 2022).

Ethical and Regulatory Considerations: Clinical trials in neurological disorders are inherently complex and expensive, involving rigorous ethical oversight and adherence to stringent regulatory guidelines. The design of trials to detect subtle but clinically meaningful improvements in neurological function can be challenging, requiring large sample sizes and long follow‐up periods (Och et al. 2022; Ye et al. 2021).

Methodological limitations of current clinical trials: While promising, the existing clinical trials on BBR in neurological conditions are often limited by small sample sizes, short durations, or specific patient cohorts (e.g., acute ischemic stroke). There is a need for larger, multi‐center, placebo‐controlled, double‐blind randomized clinical trials with clearly defined neurological endpoints and long‐term follow‐up to definitively establish BBR's efficacy and safety in various age‐related neurological disorders (Ye et al. 2021; Ju et al. 2018).

Therefore, while the preclinical evidence for BBR's neuroprotective potential is compelling, and initial clinical data are encouraging, overcoming these translational challenges is paramount for its successful integration into mainstream therapeutic strategies for age‐related neurological disorders. Future research efforts should prioritize larger, well‐designed clinical trials that address these limitations, alongside continued mechanistic studies to fully elucidate BBR's complex actions in the human brain.

## 12 Future Directions

Despite the compelling preclinical evidence and initial clinical insights into BBR's neuroprotective potential, several critical research gaps must be addressed to fully realize its therapeutic promise in age‐related neurological disorders. Future investigations should systematically focus on the following key areas:

### 12.1 Enhancing Bioavailability and Brain Permeation

#### 12.1.1 Novel Delivery Systems

While nanotechnology shows promise, further optimization of existing (e.g., specific lipid nanoparticles, polymeric micelles, exosome‐based carriers) and exploration of novel nanodelivery systems is crucial to significantly improve BBR's systemic bioavailability and its ability to consistently and effectively cross the BBB to achieve therapeutically relevant concentrations in target brain regions (Mohi‐Ud‐Din et al. 2022; El‐Nahas et al. 2023). This includes optimizing particle size, surface functionalization, and stability in biological fluids.

#### 12.1.2 Nose‐To‐Brain Delivery

Given the emerging success of nasal administration, further studies are needed to refine these methods for BBR, focusing on formulation optimization, long‐term safety, and precise brain distribution mapping in relevant animal models and, eventually, humans (Sun et al. 2025; Abo El‐Enin et al. 2022).

#### 12.1.3 Metabolic Modulation

Investigate strategies to inhibit BBR's rapid metabolism (e.g., targeting specific CYP450 enzymes responsible for its breakdown) or to enhance the conversion of BBR to more bioavailable forms like dihydroberberine (dhBBR) in vivo (Buchanan et al. 2018; Wang, Feng, et al. 2017).

### 12.2 Elucidating Mechanisms With Greater Specificity

#### 12.2.1 Dose–Response and Time‐Course Studies

Conduct more comprehensive in vivo studies across various neurological disease models (e.g., AD, PD, HD, MS, stroke) to establish optimal therapeutic dosing regimens and intervention windows (e.g., pre‐symptomatic, early‐symptomatic, advanced disease) for BBR. This should also include evaluating long‐term effects (Cameron et al. 2008; Zhao, Lu, et al. 2021).

#### 12.2.2 Targeted Mechanistic Validation

While broad pathways (oxidative stress, inflammation) are known, future research needs to pinpoint the *specific* molecular targets and downstream effectors of BBR within distinct neuronal and glial cell types relevant to each neurological disorder. For instance, detailed studies on its interaction with specific protein aggregates (Aβ, tau, α‐synuclein, mHTT) beyond general autophagy induction are warranted.

#### 12.2.3 Role of Gut Microbiota

Rigorous preclinical and clinical studies are needed to confirm the precise contribution of gut microbiota modulation to BBR's neuroprotective effects. This includes identifying specific microbial species or metabolites influenced by BBR that exert neurobeneficial actions and exploring the therapeutic potential of BBR‐microbiota interaction as a novel intervention strategy (Habtemariam 2020; Zhang, Wu, et al. 2021).

### 12.3 Comparative Studies and Combination Therapies

Evaluate BBR's efficacy and safety in head‐to‐head comparisons with established neuroprotective agents or standard‐of‐care treatments where applicable.

Explore the potential for BBR as an add‐on therapy or in combination with other compounds to achieve synergistic neuroprotective effects, potentially allowing for lower doses and reduced side effects.

By addressing these specific research directions, the scientific community can move towards a more definitive understanding of BBR's therapeutic potential in age‐related neurological disorders and facilitate its eventual clinical application.

## 13 Conclusions

Currently, numerous studies indicate the potential of oral BBR supplementation to offer protective properties for the brain in both chronic neurodegenerative diseases and acute brain injury stemming from ischemia–reperfusion or specific drug exposures. Multiple preclinical and some nascent clinical investigations have shown promising results of oral BBR supplementation in shielding against progressive neurological disorders and sudden brain damage. However, a significant concern regarding its effectiveness in clinical settings is its limited oral bioavailability, typically less than 1%. Experts have indeed observed lower levels of BBR in the brain compared to other parts of the body following oral consumption. This observation has raised the intriguing possibility that the gut microbiota, which has been increasingly associated with various neurological conditions, could significantly contribute to BBR's neuroprotective benefits, a hypothesis that warrants further clinical validation.

In summary, the positive influence of BBR on the nervous system can be attributed to its diverse effects on multiple targets. These include its well‐documented antioxidant, anti‐apoptotic, anti‐necroptotic, and anti‐inflammatory properties, as well as its ability to induce autophagy and modulate the actions of CYP450 enzymes and gut microbiota. Furthermore, to overcome BBR's limited absorption in medical scenarios, the development of nanodrug delivery systems has proven to be a crucial and promising approach. These systems, encompassing polymeric, lipid, graphene, silver, dendrimer, magnetic mesoporous silica, and gold nanoparticles, have made significant progress in recent times. Notably, nanotechnology, particularly in nasal drug delivery, has become increasingly popular in improving the effectiveness of BBR in treating neurological disorders by enhancing its brain targeting and bioavailability.

## Author Contributions

**Xiaolan Wang:** conceptualization (equal), data curation (equal), investigation (equal), methodology (equal), validation (equal), visualization (equal), writing – original draft (equal), writing – review and editing (equal). **Ruiliang Hou:** conceptualization (equal), data curation (equal), investigation (equal), methodology (equal), validation (equal), visualization (equal), writing – original draft (equal), writing – review and editing (equal). **Zhihao Chen:** conceptualization (equal), data curation (equal), investigation (equal), methodology (equal), methodology (equal), validation (equal), validation (equal), visualization (equal), visualization (equal), writing – original draft (equal), writing – review and editing (equal). **Xiaoyang Wang:** conceptualization (equal), data curation (equal), investigation (equal), methodology (equal), validation (equal), visualization (equal), writing – original draft (equal), writing – review and editing (equal). **Melika Malek:** conceptualization (equal), data curation (equal), investigation (equal), methodology (equal), supervision (equal), validation (equal), visualization (equal), writing – original draft (equal), writing – review and editing (equal). **Haoyu Wang:** conceptualization (equal), data curation (equal), investigation (equal), methodology (equal), validation (equal), visualization (equal), writing – original draft (equal), supervision (equal with Melika Malek), writing – review and editing (equal).

## Ethics Statement

The authors have nothing to report.

## Consent

The authors have nothing to report.

## Conflicts of Interest

The authors declare no conflicts of interest.

## Data Availability

Data sharing not applicable to this article as no datasets were generated or analyzed during the current study.
